# Multi-Angle Effector Function Analysis of Human Monoclonal IgG Glycovariants

**DOI:** 10.1371/journal.pone.0143520

**Published:** 2015-12-11

**Authors:** Tetyana Dashivets, Marco Thomann, Petra Rueger, Alexander Knaupp, Johannes Buchner, Tilman Schlothauer

**Affiliations:** 1 Biochemical and Analytical Research, Large Molecule Research, Roche Pharma Research and Early Development (pRED), Roche Innovation Center, Penzberg, Germany; 2 Pharma Biotech Development Penzberg, Roche Diagnostics GmbH, Penzberg, Germany; 3 Center for Integrated Protein Science Munich, Department Chemie, Technische Universität München, 85748, Garching, Germany; King's College London, UNITED KINGDOM

## Abstract

Therapeutic performance of recombinant antibodies relies on two independent mechanisms: antigen recognition and Fc-mediated antibody effector functions. Interaction of Fc-fragment with different FcR triggers antibody-dependent cellular cytotoxicity and complement-dependent cytotoxicity and determines longevity of the antibody in serum. In context of therapeutic antibodies FcγRs play the most important role. It has been demonstrated that the Fc-attached sugar moiety is essential for IgG effector functionality, dictates its affinity to individual FcγRs and determines binding to different receptor classes: activating or inhibitory. In this study, we systematically analyze effector functions of monoclonal IgG1 and its eight enzymatically engineered glycosylation variants. The analysis of interaction of glycovariants with FcRs was performed for single, as well as for antigen-bound antibodies and IgGs in a form of immune complex. In addition to functional properties we addressed impact of glycosylation on the structural properties of the tested glycovariants. We demonstrate a clear impact of glycosylation pattern on antibody stability and interaction with different FcγRs. Consistent with previous reports, deglycosylated antibodies failed to bind all Fcγ-receptors, with the exception of high affinity FcγRI. The FcγRII and FcγRIIIa binding activity of IgG1 was observed to depend on the galactosylation level, and hypergalactosylated antibodies demonstrated increased receptor interaction. Sialylation did not decrease the FcγR binding of the tested IgGs; in contrast, sialylation of antibodies improved binding to FcγRIIa and IIb. We demonstrate that glycosylation influences to some extent IgG1 interaction with FcRn. However, independent of glycosylation pattern the interaction of IgG1 with a soluble monomeric target surprisingly resulted in an impaired receptor binding. Here, we demonstrate, that immune complexes (IC), induced by multimeric ligand, compensated for the decreased affinity of target bound antibody towards FcRs, showing the importance of the IC-formation for the FcR- mediated effector functions.

## Introduction

Over the past several years, therapeutic antibodies for the treatment of various diseases have become a considerable part of the biopharmaceutical industry. More than 40 therapeutic mAbs and mAb fragments are approved and prescribed today [1,2], the majority being of the IgG isotype.[3] The IgG molecule consists of two light chains (two domains each) and two heavy chains (four domains each). Each light chain together with two domains of a heavy chain forms the Fab (fragment antigen binding) region. Both Fabs are linked via a flexible hinge to the Fc (fragment crystallizable) region, formed by the dimer of the remaining domains of the two heavy chains.

The clinical efficacy of therapeutic antibodies rely on two functional properties: first, the ability of the Fab regions to specifically recognize and bind the target; and second, the ability to induce different immune system effector mechanisms through interaction of the Fc region with Fc gamma receptors (FcγRs), the C1q component of complement and the neonatal receptor (FcRn).

In the context of therapeutic antibodies, Fc gamma receptors play the most prominent role in induction of effector mechanisms. Human FcγRs are divided into the three main groups I, II and III; in addition the FcγRII and FcγRIII subgroups are comprised of IIa, IIb, IIc and IIIa, IIIb, respectively. FcγRs differ in affinity for the antibody Fc-part. The FcγRI is usually described as a high affinity receptor and was shown to bind both single IgGs and immune complexes [4], whereas the FcγRII and the FcγRIII require immune complexes to elicit effector functions, and are referred to as receptors with low and moderate affinity respectively. In terms of initiated immune response, FcγRs can be further classified as activating or inhibitory. The inhibitory receptors are represented by one FcγR—FcγRIIb. The rest are activating receptors. Following binding to an IgG or immune complexes activating receptors induce: antibody dependent cell-mediated cytotoxicity (ADCC), phagocytosis and endocytosis, promote antigen presentation and release of pro-inflammatory mediators. The receptor FcγRIIb modulates the immune response by inhibiting the ability of activation of activating receptors to activate effector cells. Thus, immune responses are largely tuned by the balance between activating and inhibitory functions of the FcγRs (reviewed in refs [5–10]).

**Fig 1:**
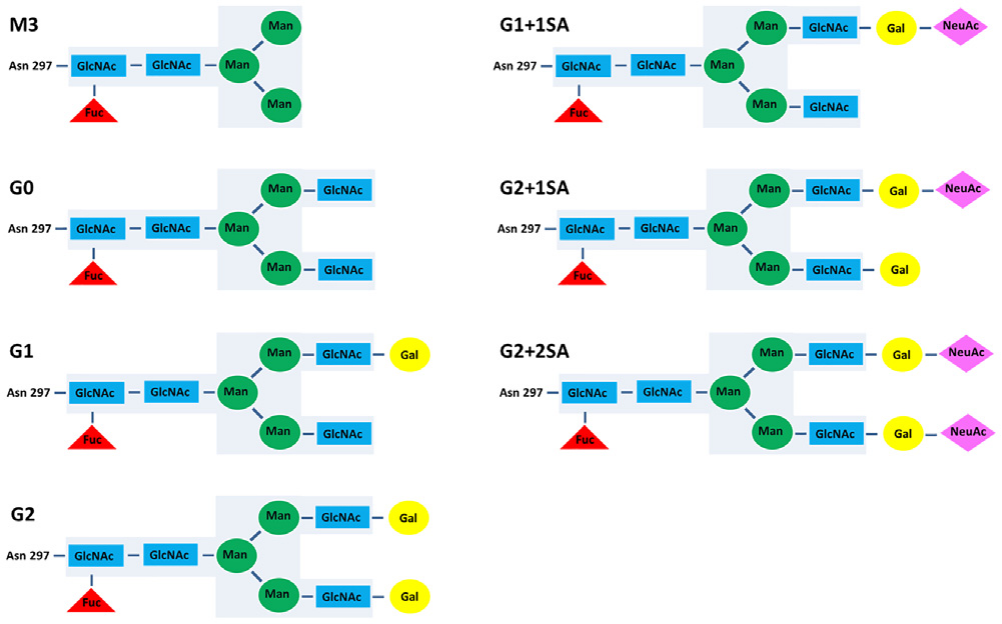
Structures of IgG N-glycans attached to the Asn297 in the Fc domain. The core (G0) heptasaccharide is highlighted in light blue. GlcNAc, N-acetylglucosamine; Fuc, fucose; Man, mannose; Gal, galactose; NeuAc, N-acetyl neuraminic (sialic) acid.

In addition to immune mechanisms induced by interactions with FcγRs, binding of the antibody Fc-part to the C1q component initiates the classical complement activation pathway leading to complement dependent cytotoxicity (CDC). Interaction with the FcRn protects IgGs from degradation and is crucial for the turnover and half-life of antibodies in serum. In addition, FcRn was reported to regulate the intracellular sorting of IgG immune complexes for the subsequent antigen presentation.[11–14] It has long been recognized, that the IgG-associated glycan plays an important role in the interaction of antibodies with Fc-receptors and complement activation, and thus is crucial for the antibody effector function.[15–18] In IgGs, a sugar moiety is linked to the Asn297 of the heavy chain in the Fc region. The Fc-attached oligosaccharide is characterized by heterogeneity and essentially consists of a biantennary core heptasaccharide, comprised of N-acetylglucosamine and mannose, which can be varied by the addition of bisecting N-acetylglucosamine, galactose, fucose, and sialic acid (Fig 1). An IgG with no galactose residue on the core sugar is referred to as a G0 glycoform, the presence of terminal galactose residue on one or two branches of sugar moiety define the G1 and G2 glycoforms, respectively. G1 and G2 can be further modified by the addition of sialic acid (SA) residues linked to the respective galactose, resulting in G1+1SA, G2+1SA and G2+2SA glycoforms. Bisecting N-acetylglucosamine and “core fucose” (fucose residue in an α1,6-linkage to the first GlcNAc) also contribute to the variety of the IgG-attached glycan. It should also be mentioned, that the two heavy chains of the same IgG molecule may carry different sugar moieties.[19,20]

It has been demonstrated that glycosylation is essential for IgG functionality, and that the composition of the attached oligosaccharide dictates affinity of the antibody for individual FcγRs and determines binding to different FcγR classes: activating or inhibitory.[19,21–23]

Core fucosylation has by far the greatest impact on binding to FcγR and is the most crucial parameter, when antibodies with increased ADCC activity are desired. Absence of the core fucose was demonstrated to result in an up to 50-fold increase of antibody affinity to FcγRIIIa and FcγRIIIb, leading to enhanced ADCC activity.[24–27] Terminal galactose residues are reported to enhance CDC activity as result of improved C1q binding[28], and to improve interaction with FcγRs[29,30] (Tejada et. al., manuscript in preparation). Recent studies suggest that antibodies with high sialic acid content play an important role in anti-inflammatory responses. It has been reported that interaction of highly sialylated antibodies with the C-type lectin dendritic cell-specific intercellular adhesion molecule (ICAM)-grabbing non-integrin (DC-SIGN) triggers an anti-inflammatory pathway, resulting in up-regulation of the inhibitory FcγRIIb and down-regulation of the activating receptors.[31,32] Additionally, Fc-glycans bearing terminal sialic acid residues were reported to exhibit a decreased affinity for activating FcγRs, for example FcγRIIIa, leading to a reduced ADCC induction.[33,34] However, in another report, sialic acid was demonstrated to either not effect or to improve antibody interaction with FcγRs [35]

In addition to functional aspects, Fc glycan pattern has been demonstrated to have an impact on the conformational and structural properties of the antibody Fc, ensuring appropriate interaction with FcγRs. [36,37] Glycosylation has also been shown to play an important role in maintaining the structural integrity, stability and solubility of IgGs.[38–40]. For several mAbs it has been shown that deglycosylation as well as the glycosylation results in a loss of effector function—an inability of the respective antibodies to induce either CDC or ADCC.[41,42] Taken together these findings highlight the importance of the Fc-attached oligosaccharide and its composition for antibody structure and effector functions.

In addition to the glycosylation pattern, the size of the immune complex also contributes to the induction of effector mechanisms. As mentioned above, FcγRI is the only receptor demonstrating high affinity interactions for a single IgG; the remaining FcγRs are only fully activated by IgGs in multimeric immune complexes (ICs). The size of ICs is determined by the abundance or availability of the antigen and antibody, and may have a crucial impact on interactions with cellular FcγRs. Lux et al.,[43] for example, report an improved binding of multimeric immune complexes to the FcγRs, and that binding of the EndoS deglycosylated IgGs to the affinity FcγRs was not abolished, when these IgGs were a part of an IC. In addition, binding of the antibody to antigen was reported to affect changes in the conformation of the Fc-region, which may impact antibody interactions with Fc-receptors. The size of the antigen could also have an influence on antibody—receptor interactions. [44,45] Thus it is important to address the interaction of antibodies with Fc-receptors in the context of target binding and/or as a part of an immune complex.

In this work we set out to address antibody-mediated effector functions of IgG1 and its eight enzymatically engineered glycovariants: deglycosylated, M3, G0, G1, G1+1SA, G2, G2+1SA and G2+2SA. The addressed monoclonal IgG1 against a receptor of the EGFR family was expressed in CHO cells, exhibits effector function and has ADCC as part of its mechanism of action. As induction of different effector mechanisms requires binding of antibodies to the respective Fc-receptors, we examined the interaction of the above glycovariants in presence or absence of antigen with the Fc-gamma receptors and FcRn.

## Results

### Stability and aggregation propensity of IgG1 glycovariants

The tested glycovariants: deglycosylated, M3, G0, G1, G1+1SA, G2, G2+1SA and G2+2SA (Fig 1) were generated by *in vitro* glyco-engineering as described by Thomann et al.,[46] or in Material and method section. Application of a novel technique enabled production of antibodies with a purity for a respective glycan of up to 85%. Wild type (WT) antibody (not glycoengineered) was used as reference. To the best of our knowledge, structural and functional features of glycosylation variants of such quality and composition have never been analyzed before side by side.

**Fig 2:**
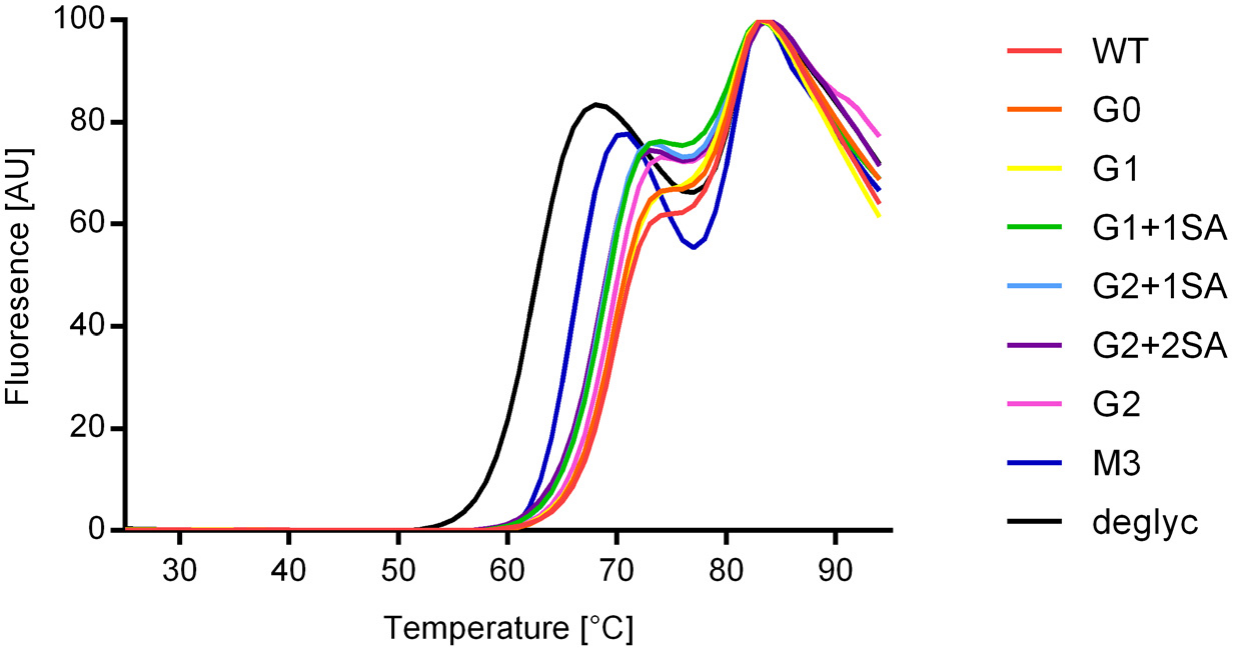
Thermal transitions of IgG1 glycovariants obtained by Thermofluor Stability Assay (TSA). Melting curves: deglycosylated (black), M3 (blue), G0 (green), G1 (pink), G2 (orange), G1+1SA (light blue), G2+1SA (yellow), G2+2SA (purple), WT (red).

As glycosylation has been reported to be important for the structural integrity and stability of antibodies[47–50], we assessed the thermal stability of the samples by applying the Thermofluor Stability Assay (TSA) using the fluorescent dye SYPRO orange. SYPRO orange interacts with the hydrophobic stretches of a polypeptide chain, leading to an increase in fluorescence. This effect is exploited to monitor thermal denaturation, when the hydrophobic core residues become solvent-exposed upon unfolding.[51,52] SYPRO orange was added to the samples which were then heated from 25°C to 95°C. Thermal unfolding of antibodies is a multistep process comprising several transitions. [53] The first transition peak corresponds to the unfolding of the C_H_2 domains, the following transitions to the unfolding of the Fabs and the C_H_3 domains, respectively. However, the final transitions can merge into one single peak. [54] Analysis of the thermal stability profiles of IgG1 glycovariants revealed two transitions for all antibodies tested. Comparison of melting temperatures (midpoint of the respective transition), showed no differences in the second transition for all glycovariants, with a melting temperature of ~80°C (Fig 2, Table 1), ruling out the influence of the sugar pattern on the stability of C_H_3 and the Fab region. Interestingly, there were differences in the unfolding of the C_H_2 domain. The M3 variant and the deglycosylated antibody demonstrated a shift to lower melting temperatures of 65°C and 61.5°C respectively, compared to 68–69°C obtained for the other glycovariants. This suggests that the glycosylation pattern plays a role in sustaining the structural stability of the C_H_2 domain.

**Table 1:** Melting points of the two thermal transitions of IgG1 glycovariants, obtained by TSA.

Sample	Tm 1 [°C]	Tm 2 [°C]
**WT**	**69.0**	**80.0**
**G0**	**69.0**	**80.0**
**G1**	**69.0**	**80.0**
**G1+1SA**	**68.0**	**80.0**
**G2**	**68.0**	**80.0**
**G2+1SA**	**68.0**	**80.0**
**G2+2SA**	**68.5**	**80.0**
**M3**	**65.0**	**80.0**
**deglyc**	**61.5**	**79.5**

Tm = melting temperature

To examine the influence of attached sugars on the aggregation propensity of IgG1 variants, antibodies were incubated at 37°C for 10 days at pH 7.4 (physiological pH condition) and pH 6.0 (storage buffer). Subsequently, SEC was used to determine the formation of aggregates (HMW—high molecular weight species), fragmentation (LMW—low molecular weight species) and monomeric species.

At both pH conditions, minor but detectable formation of oligomeric species could be observed for all glycovariants in a range of 2–3% (S1 Fig). Thermal stress did not cause any significant antibody fragmentation and no major loss of the monomeric fraction could be observed for any of the tested glycovariants.

In summary, the deglycosylated and M3 variants tested here are less thermally stable, compared to the other glycovariants, in agreement with previously reported results. [55–57]

### Interaction of IgG1 Glycovariants with FcγRs

To address Fc-mediated functional properties of the IgG1 glycovariants, the interaction between the IgG1 glycovariants and the FcγRs was analyzed by surface plasmon resonance (SPR). In the first set of experiments the His-tagged Fc-gamma-receptors (FcγRI, FcγRIIa, FcγRIIb and FcγRIIIa) were captured via an Anti-His antibody immobilized on the chip surface. Subsequently, the IgG1 glycovariants, in equal concentrations, were injected as analytes. In the classical approach to determine the 1:1 affinity of the reference antibody to the His-captured FcγRI, FcγRIIa, FcγRIIb and FcγRIIIa, we faced the problem that none of these interactions could be saturated by 8000 nM of the IgG1 molecule in solution. This precluded an appropriate read out parameter for a precise comparison of the glycovariants, because the variability of an imprecise affinity determination would incorporate a larger variability compared to the existing differences in affinity. This is in accordance to the observations by Hayes et al [58,59]. The authors of this publication described that the heterogeneity of the receptor glycosylation such as the heterogeneity of the antibody glycosylation reasons the challenge of rate and affinity constants determination. To exemplify the problem we added such an affinity determination in S2B and S2C Fig. To cope with the affinity determination problem we developed a non-kinetic comparative analysis to quantify the binding of the tested variants to the FcγRs. We have determined this read out point out of a steady state analysis and set the applied antibody concentration for the respective Fc receptor interaction to the inflection point of the dose response curve. Each receptor has been captured every cycle in the same concentration and also the antibody analyte solution was set to a standard concentration for each receptor class (FcγRI = 100nM, FcγRIIa/b = 300 nM and FcγRIIIa = 200 nM). Binding of the WT IgG1 was set as 100%. Since the absence or presence of the core fucose is crucial for the interaction with FcγRIIIa, it is worth mentioning, that all test glycovariants have similar level of afucosylation of about 7–9%.

**Fig 3:**
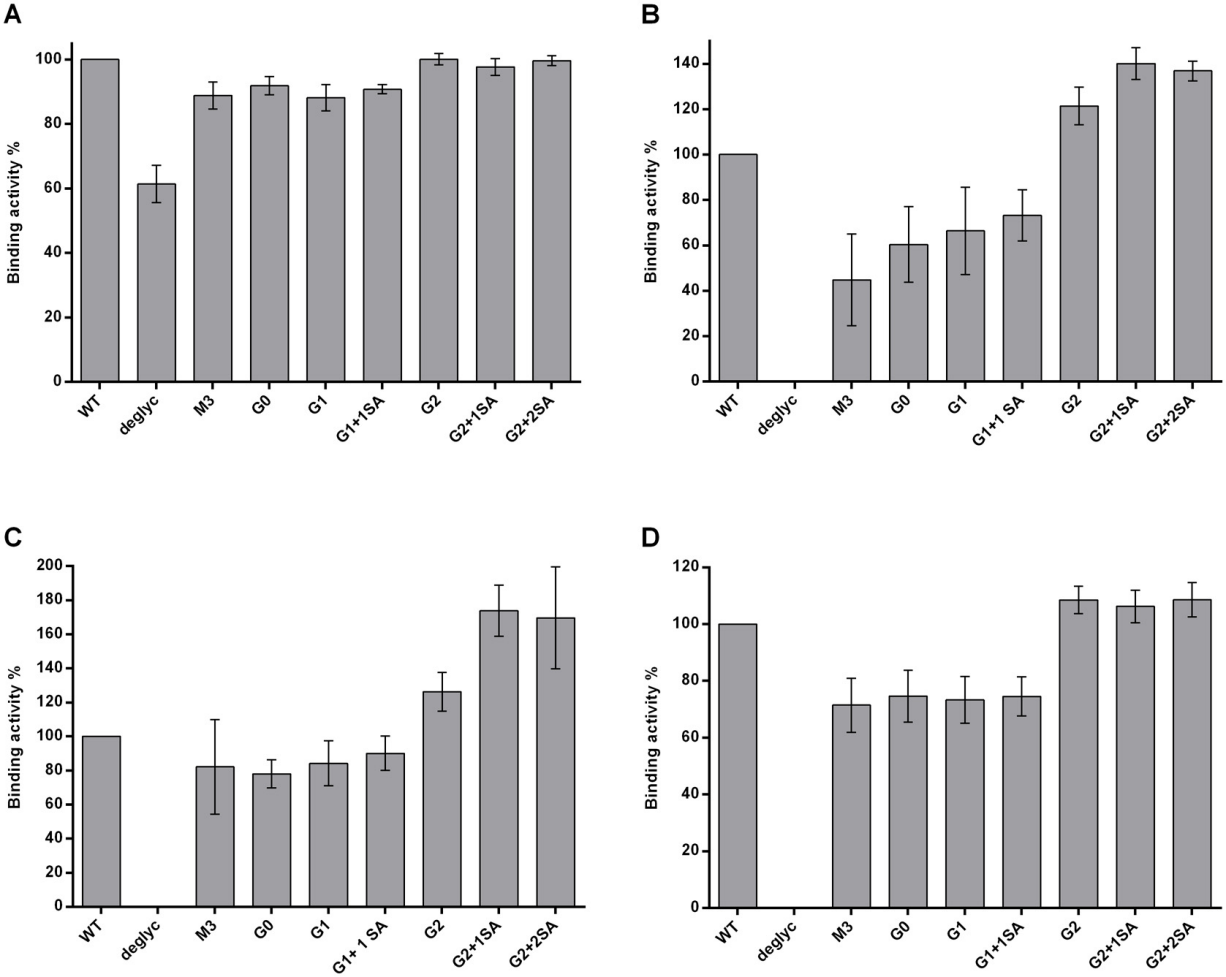
SPR analysis of interaction of IgG1 glycovariants with FcγRs. Anti His-antibody immobilized on the chip, glycovariants were injected as analytes, after capturing of the respective receptors: A. FcγRI, B. FcγRIIa, C. FcγRIIb and D. FcγRIIIa. Binding of WT antibody was set as 100%. Each graph represents results from at least three independent experiments; data are given as means ± SD.

Comparison of binding to the FcγRI showed similar interactions for all glycovariants tested, with the exception of the deglycosylated Ab, which had approximately a 40% decrease in binding (Fig 3A). Moreover, no detectable binding to either FcγRIIa and FcγRIIb or FcγRIIIa could be measured for this glycovariant. G0, G1 and G1+1SA interact with FcγRIIa with binding efficiencies of 65–73%, and M3 demonstrated binding of about 45%, compared to the WT (Fig 3B). The M3 glycovariant also had the weakest binding to FcγRIIb and FcγRIIIa of about 65% and 70% respectively (Fig 3C and 3D). FcγRIIb interaction with G0, G1 and G1+1SA ranged from 80% to 90%, and FcγRIIIa demonstrated binding activity of about 75% for these glycovariants. Bi-galactosylated antibodies (G2, G2+1SA and G2+2SA) appear to have an increased binding to FcγRIIa, FcγRIIb and FcγRIIIa (106–160%). Sialylation doesn’t seem to impair binding to FcγRs, on the contrary, FcγRIIa and FcγRIIb interaction appears to be improved for the sialic acid bearing variants. Interestingly, we didn’t observe any decreased interaction of the FcγRs with the variants terminating in sialic acid, in contrast to previously reported results.[60–62]

To rule out the possible influence of the Fab fragments on the Fc-region—FcγR interaction, the previously described experimental approach was applied to test binding of Fc-fragments isolated from the respective IgG1 glycovariants. Analysis of the results confirmed tendencies already observed for the full size IgG(S2A Fig): similar binding pattern of all variants, except the deglycosylated, to FcγRI inability of the deglycosylated antibody to bind any receptor, except for FcγRI, as well as enhanced binding efficiencies for the bi-galactosylated variants to FcγRIIa, FcγRIIb and FcγRIIIa. In other words, these results demonstrate that isolated Fc fragments retain the ability to bind FcγR with efficiency comparable to whole IgG.

**Fig 4:**
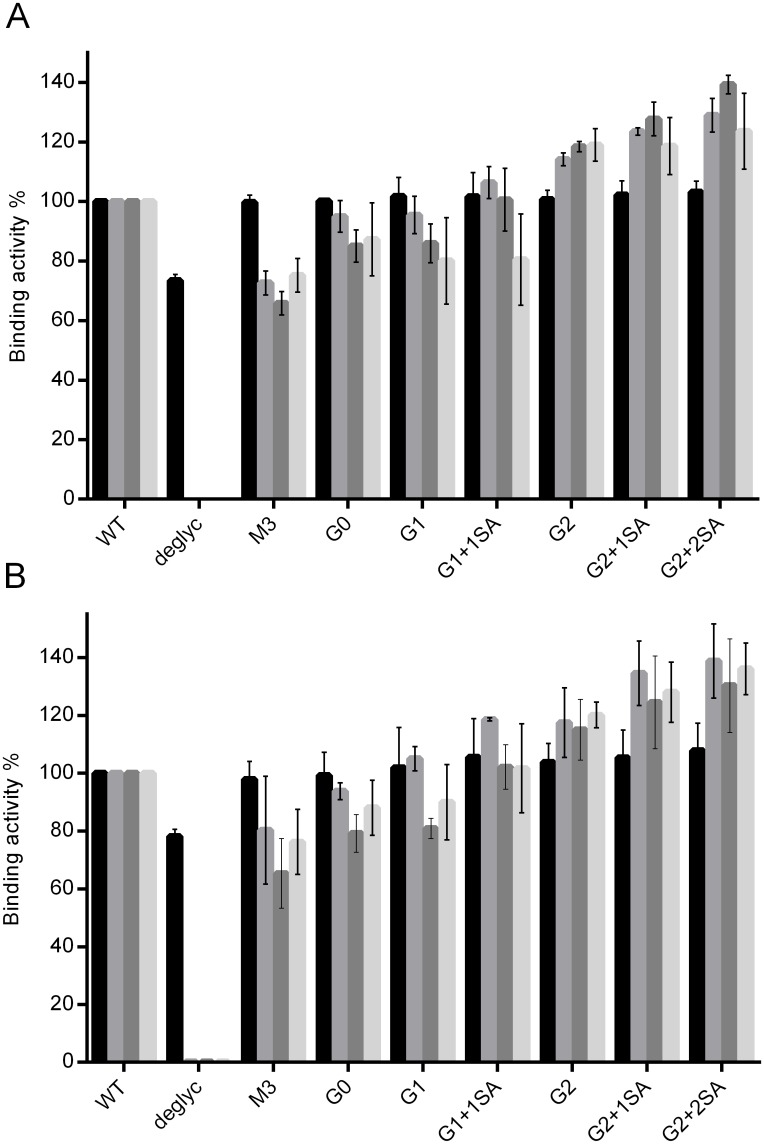
SPR analysis of the interaction of target-bound or F(ab’)_2_ <Fab-captured IgG1 glycovariants with FcγRs. Glycovariants were injected to bind antigen (A) or F(ab’)_2_ <Fab> (B), immobilized on the chip, followed by the application of the respective receptors: FcγRI (black), FcγRIIa (grey), FcγRIIb (dark grey) and FcγRIIIa (light grey). Binding of WT antibody was set as 100% Each graph represents results from at least three independent experiments; data are given as means ± SD.

Binding of the antibody to its target was reported to induce conformational changes and influence antibody interaction with the Fc-receptors. To address this issue, binding of the FcγRs to the antigen-bound glycovariants was tested. For that, the IgG1 target was immobilized on the chip surface. IgG1 glycovariants were bound onto the antigen-covered surface, followed by the injection of the various FcγRs as analytes (FcγRI, FcγRIIa, FcγRIIb and FcγRIIIa) (Fig 4A). Analogous to previous results FcγRI binding was comparable for all tested glycoforms, except for the deglycosylated form. Minimal differences were observed for the interaction of G0, G1 and G1+1SA with FcγRIIa, FcγRIIb and FcγRIIIa, with binding efficiencies slightly higher than those of antigen free antibodies, in the range of 70–90%, in comparison to the reference antibody. Again, the binding efficiencies of bi-galactosylated variants were set apart, showing improved interaction of 110–150%. Also in this case, sialylation does not seem to hinder receptor binding.

To determine if the improved antibody—receptor interactions were due to antigen binding, and not merely caused by antibody orientation, the analyzed glycovariants were captured by anti-Fab F(ab’)_2_ immobilized on the chip surface; followed by the application of the FcγRs. This SPR-assay confirmed the already obtained interaction pattern of glycovariants with FcγRs (Fig 4B). The assay using immobilized anti-Fab F(ab’)_2_ demonstrated an overall increase in binding activities, compared to the initial assay with the captured receptor.

### Analytical FcγRIIIa affinity column

SPR is a potent and conventional method, applied to address antibody—receptor interaction. However, analysis of such interaction for complex samples is challenging; as this method doesn’t distinguish between the interactions of different species in the sample. As next step, analytical FcγRIIIa affinity chromatography provides a possible solution to this problem. Here, human FcγRIIIa is immobilized on the column; antibody samples are loaded onto the column and eluted by linear pH gradient from 6.0 to 3.0. Stronger antibody-receptor interactions correlate with longer retention times. As reported by Thomann et al.,[46] afucosylated antibodies (with 50 fold higher affinity towards FcγRIIIa) analyzed with the FcγRIIIa-column demonstrated longer retention times, compared to the fucosylated IgGs.

**Fig 5:**
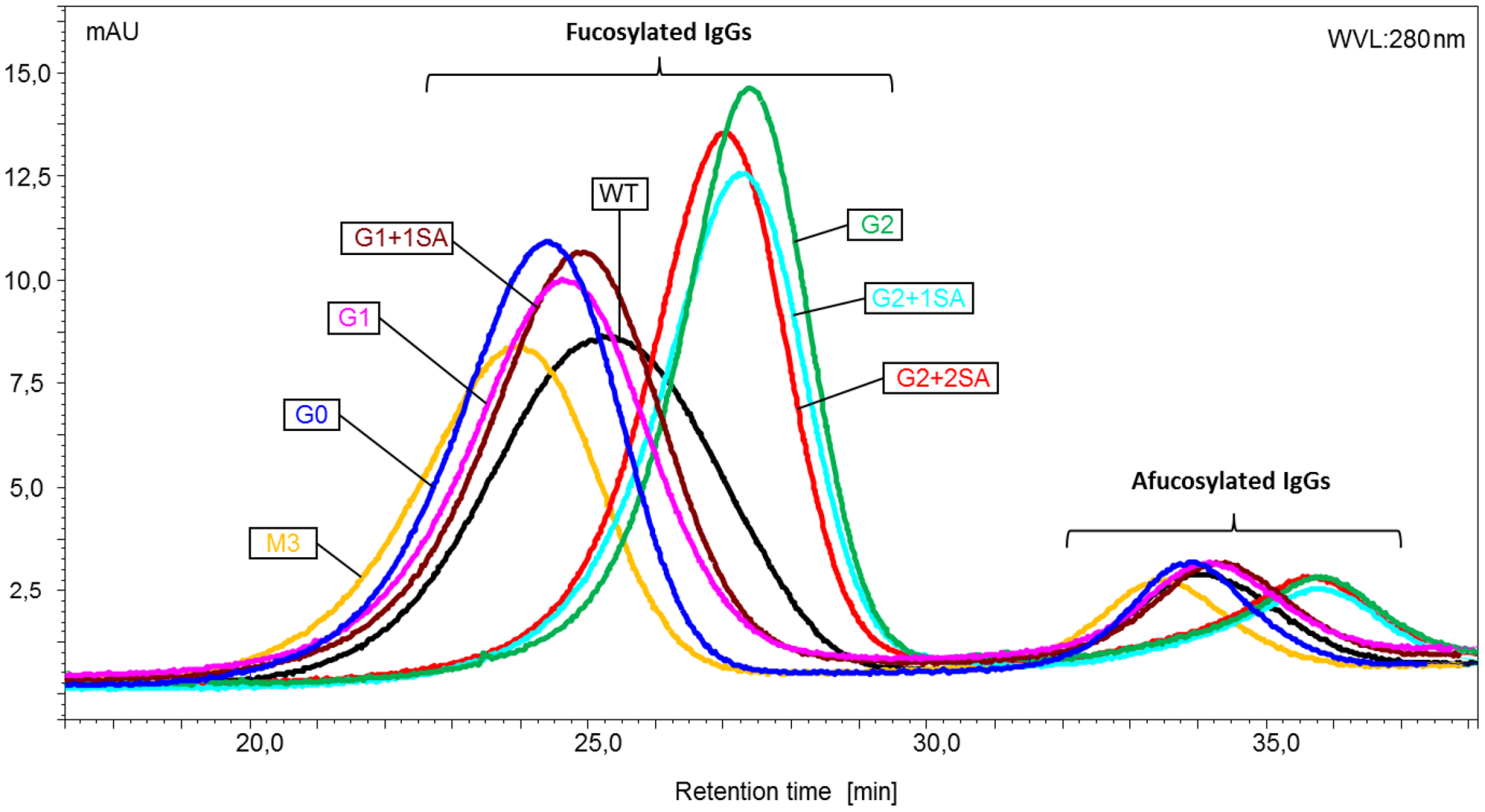
Retention profiles of the IgG1 glycovariants obtained by analytical FcγRIIIa affinity chromatography. The chromatograms, obtained by monitoring signal at 280 nm, show two peaks corresponding to fucosylated and afucosylated antibody fractions (deglycosylated IgG is not shown).

Consistent with our previous results, the deglycosylated variant showed no binding to the FcγRIIIa receptor, and eluted immediately upon loading onto the column. The retention profiles of the remaining glycovariants showed two elution peaks, corresponding to two antibody species: fucosylated (main peak with retention times in the range of 24–26 min) and afucosylated -small peak with retention times between 33 and 36 min, which is in line with the afucosylation level of about 7–9% for all glycovariants analyzed (Fig 5). The distribution of the retention times was similar to the binding pattern, obtained by SPR analysis. That is: M3 < G0 < G1 < G1+1SA < WT (Fig 5). Bi-galactosylated variants, independent of the presence of sialic acid, showed a shift to longer retention times and thus stronger binding. The fraction of afucosylated antibodies showed delayed retention times, confirming stronger binding. Interestingly, the retention pattern of afucosylated antibodies strongly resembles that of the fucosylated variants, suggesting that along with the major impact of afucosylation, receptor interaction is still influenced by the remaining sugars.

### Interaction of IgG1 glycovariants with FcγRIIIa on the cell surface

Analysis of binding activities of IgGs to FcγRs by SPR or FcγRIIIa affinity column provides insight into antibody receptor interaction; however both methods focus on the interaction of isolated proteins and might not depict the interaction as it occurs at the cellular level. Therefore, we employed a cell-based Fluorescence Resonance Energy Transfer (FRET) to examine the interaction of the IgG1 glycovariants with FcγRIIIa expressed on the surface of live cells. FRET is based on the transfer of energy between two fluorophores, a donor and an acceptor, when in close proximity. Here, proteins of interest are fused with a SNAP-tag, which can covalently interact with fluorescent dyes (donor or acceptor). The FcγRIIIa receptor cellular binding assay is a competitive assay, which uses cells expressing a specific FcγRIIIa variant conjugated to Lumi4-terbium cryptate (donor), and human IgG1 labeled with d2-dye (acceptor). The unlabeled analyte antibody competes with d2-IgG1 for binding to the receptor, resulting in a decrease in the FRET signal

**Fig 6:**
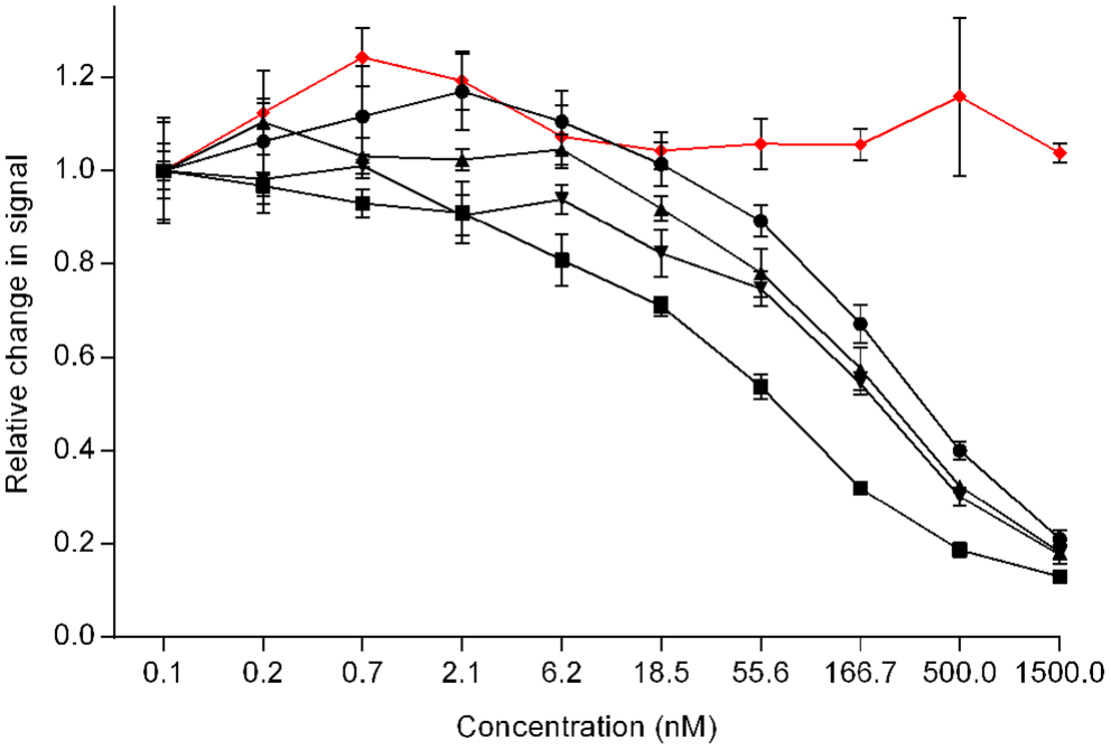
Binding of glycovariants to FcγRIIIa, expressed on living cells. Unlabeled glycovariants compete for binding to receptor with acceptor-labeled antibody, resulting in decrease of FRET signal: deglycosylated (red diamonds), WT (black circles), G0 (black triangles), G1(black inverted triangles), G2 (black squares). Initial signal was normalized to 1. (For remaining glycovariants refer to S3 Fig).

The obtained results demonstrate a clear decrease in FRET signal for all tested glycovariants, with the exception of the deglycosylated antibody (Fig 6, S3 Fig). Consistent with our previous results, the deglycosylated IgG1 failed to bind FcγRIIIa receptor, so there is no decrease in fluorescence signal. The G2 sample showed a slightly faster decrease in signal, and thus increased receptor interaction, compared to the rest of the tested glycovariants, which is in line with our previous results.

### Interaction of target bound IgG and ICs with FcγRIIIa

Antigen bound IgG1 was demonstrated to have an enhanced binding to FcγRs, when analyzed by SPR. We employed the FcγRIIIa affinity column to further analyze binding of the target bound IgG1 glycovariants to FcγRIIIa. The antibodies were pre-incubated with the target (extracellular domain of a receptor of the EGFR family, of ∼70 kDa) in ratios of 1:1, 1:2 and 1:4. Prior to loading onto the FcγRIIIa-column, antibody-antigen complexes were analyzed by Size Exclusion Chromatography (SEC). The analysis revealed three different antibody species: antigen free antibodies and antibodies with either one or two Fabs occupied by antigen (S4 Fig). For the equimolar antibody and antigen ratio all three species were observed at comparable levels. Incubation of antibody with the twofold molar excess of antigen resulted in mono- and bivalently bound antibodies, with the latter one prevailing. A fourfold antigen excess led to the formation of almost exclusively bivalently bound antibody species.

**Fig 7:**
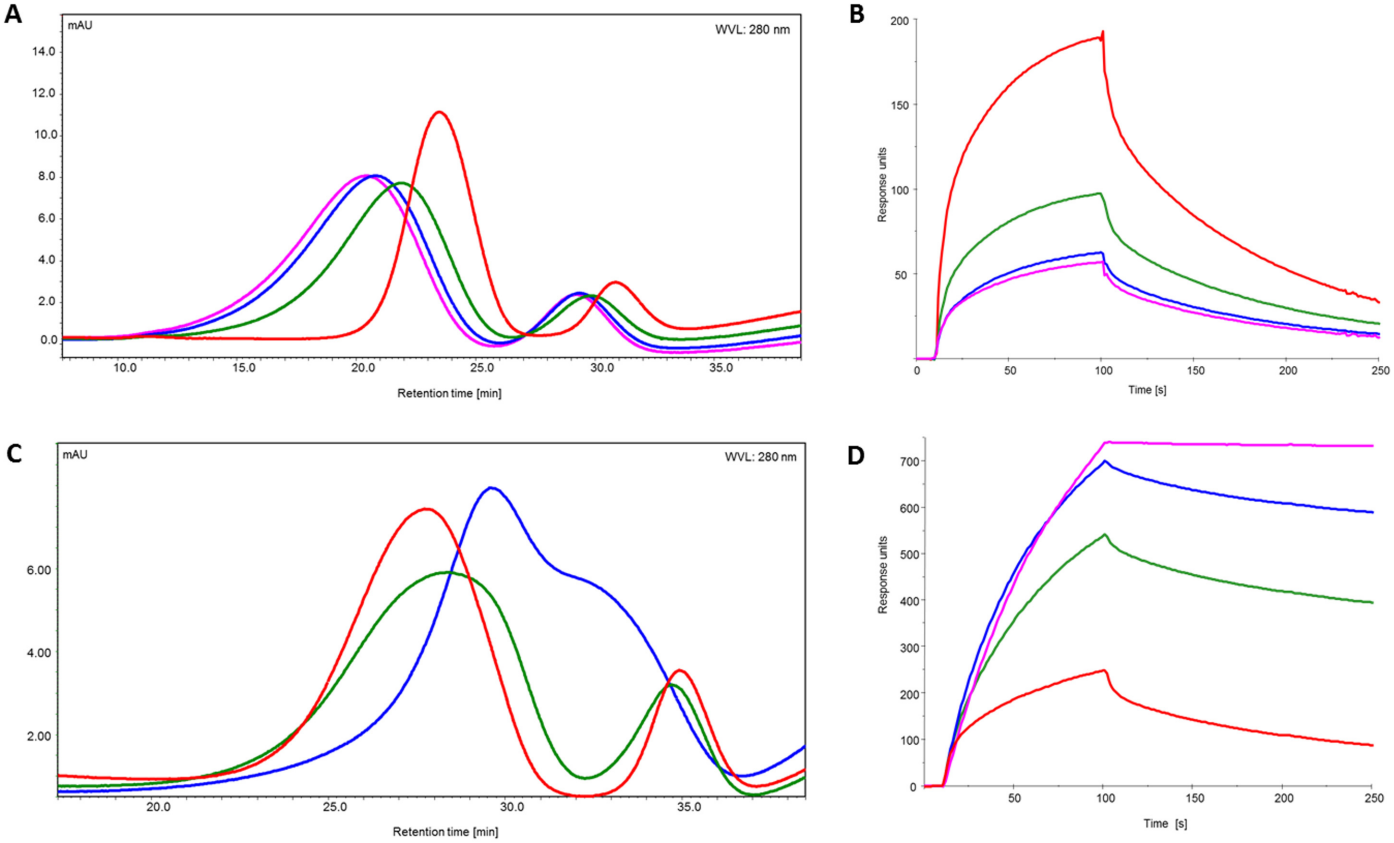
Interaction of target- or F(ab’)_2_ <Fab>-bound IgG with FcγRIIIa. Prior to interaction analysis IgG was incubated with its monomeric target (A, B) or with F(ab’)_2_ <Fab> (C, D) and loaded onto affinity column (A, C) and examined by SPR (B, D). The IgG: ligand ratio: 1:0 (red), 1:1 (green), 1:2 (blue) and 1:4 (pink).

The samples were subsequently loaded onto the FcγRIIIa column to examine receptor interaction with the target bound antibodies. Surprisingly, the analysis of the retention pattern revealed results that were against expectation. Similar to the SEC elution profile, the three different antibody species (with regard to Fab occupation by the antigen) show three different elution peaks (Fig 7A). Bivalently bound antibodies did not give stronger, as expected, but the weakest binding to the immobilized FcγRIIIa receptor, eluting first with a retention time of 20.5–21 min, followed by the fraction of monovalently bound antibody, with a retention times of ~22 min. Finally, the antigen-free antibody eluted with a retention time of 24 min. Remarkably, the afucosylated antibodies, represented by the fraction with retention times between 29 and 31 minutes, showed exactly the same differences in FcγRIIIa interaction.

In contrast to our previous results obtained by SPR, antigen-bound antibody clearly demonstrated decreased receptor interaction, compared to the antigen-free species. However, it should be pointed out that the SPR approach doesn’t fully recapitulate the experimental setup of the affinity column. To address this, we applied an SPR-method, which mimicked the setup of the affinity column. The FcγRIIIa receptor was captured, as described earlier. Then, antibody, pre-incubated with increasing amounts of antigen, was injected as analyte. Consistent with the results obtained by the means of FcγRIIIa–affinity chromatography, antigen free antibody showed the highest binding activity (Fig 7B), followed by the samples with mono- and bivalently bound antibodies.

Analysis of the antibodies bound to soluble monomeric targets revealed surprising and unexpected receptor binding behavior, i.e. binding to the soluble target caused an impaired antibody-receptor interaction. However, in the context of low and moderate affinity receptors it is important to assess the interaction with immune complexes (ICs) and not single antibodies. To form ICs, we used F(ab’)_2_ <Fab>, generated by the digestion of whole IgG by pepsin, removing the Fc-region, and leaving two Fab fragments linked by disulfide bridges in the hinge region. F(ab’)_2_ <Fab> is divalent, i.e. is able to bind both Fab fragments of the test antibody, making F(ab’)_2_ <Fab> a potent tool to mimic the interaction of IgG1 with a dimeric target. SEC analysis of IgG1 incubated in different ratios with the F(ab’)_2_ <Fab> revealed a wide range of immune complexes with molecular masses from ~700 kDa to ~4000 kDa or more, as revealed by SEC MALLS (S4B Fig). FcγRIIIa affinity column and SPR were used to examine the interaction of the ICs with the respective receptors. SPR analysis demonstrated that ICs have an up to threefold higher binding activity compared to single antibodies, independent of the glycosylation pattern (Fig 7D, S6 Fig). In line with that, the retention time of the ICs shows a distinct shift to the right (4–7 minutes), corresponding to stronger receptor binding (Fig 7C). Immune complexes formed by the introduction of F(ab’)_2_ <Fab> are larger than antibodies mono- or bivalently bound to a monomeric target, nevertheless we observed a clear shift to increasing retention time, which confirms that impaired binding of antibodies bound to monomeric target was not due to a size exclusion effect.

Additionally, SPR analysis of interaction of the F(ab’)_2_ <Fab>-linked IgGs with FcγRIIa and FcγRIIb, showed up to a 50-fold higher binding activity of the ICs to the receptors, compared to single antibodies (S6 Fig).

### Interaction of glycovariants with FcRn

In addition to Fc-mediated effector functions, interaction with FcRn (neonatal Fc receptor) is critical for the turnover and half-life of IgG antibodies in vivo. Therefore, the interaction of IgG1 glycovariants with FcRn was analyzed via SPR. In the first setup, His-tagged FcRn was captured, by the immobilized anti-His antibody, followed by injection of the glycovariants. In the second approach, FcRn was immobilized on the chip surface, followed by injection of the glycovariants. In the third approach, IgG1 glycovariants were captured via protein L (interaction with the light chain) and FcRn was loaded afterwards, and finally, FcRn interaction with target bound antibodies was analyzed.

**Fig 8:**
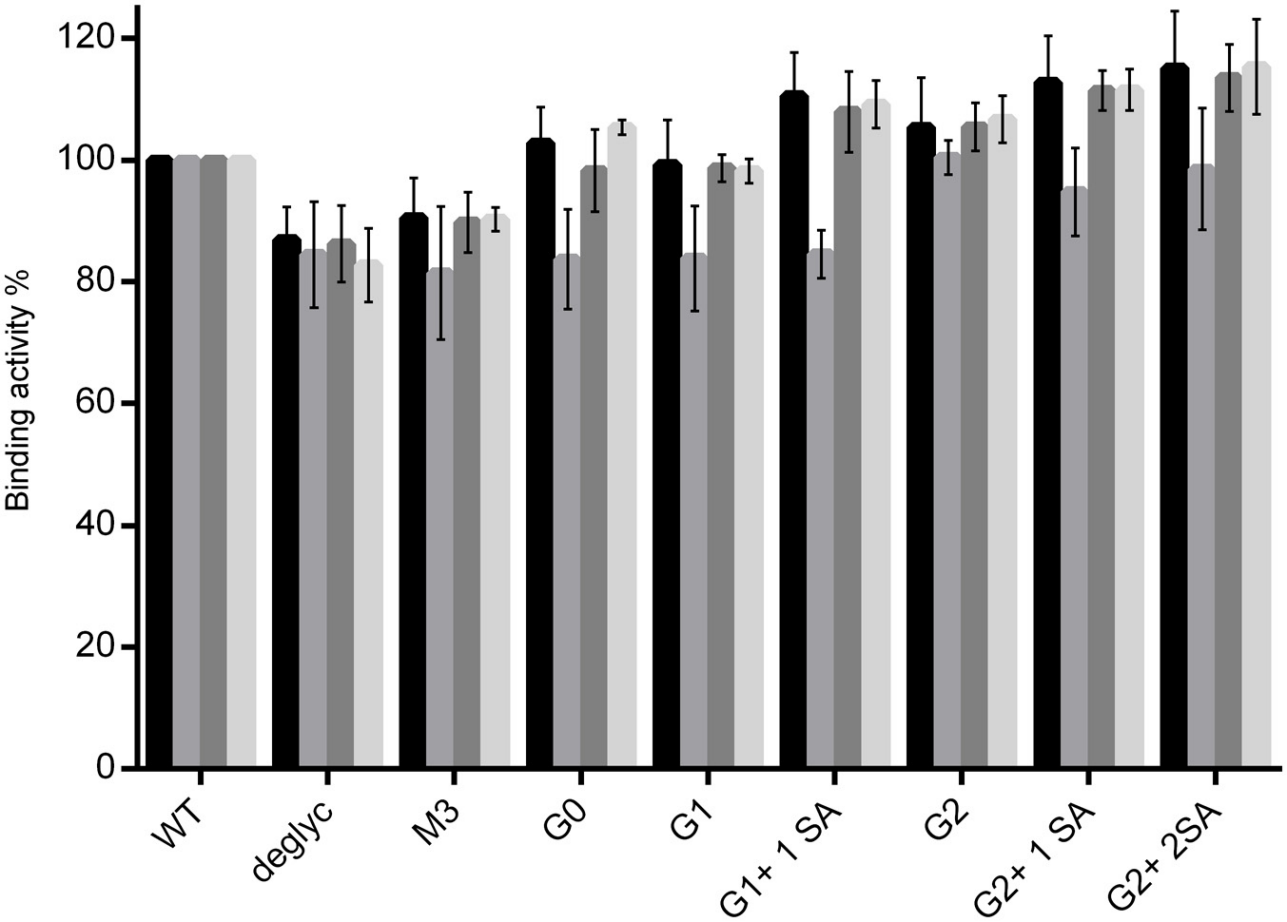
Interaction of IgG1 glycovariants with FcRn. Comparison of different SPR experimental set-ups: FcRn receptor was captured by Anti-6xHis antibody, followed by the application of IgG glycovariants (black); FcRn was directly immobilized onto the chip surface, subsequently IgG glycovariants were loaded (grey); glycovariants were captured by the immobilized protein L, followed by the application of FcRn (dark grey); glycovariants were bound onto the immobilized target, then the receptor was applied (light grey).

All setups resulted in a rather similar interaction pattern and comparison of binding activities of the glycovariants did not reveal any striking differences in the interaction with the FcRn. However, bi-galactosylated and sialylated antibodies showed slightly increased binding, whereas interaction of the M3 and deglycosylated variants was weaker than that of the other glycovariants, independent of the assay (Fig 8).

In addition to SPR analysis, the influence of glycosylation pattern on FcRn interactions was assessed by the FcRn affinity liquid chromatography method. Human FcRn was immobilized on the column and a linear pH gradient from pH 5.5 to 8.8, which resembles the physiological mechanism of the pH-dependent interaction between FcRn and IgG, was applied. Analogous to the FcγRIIIa affinity chromatography, analytical FcRn chromatography enables differentiating the interaction of IgG variants by peak characteristics and retention time profiles. FcRn affinity chromatography allowed discriminating between IgGs with different Fabs, oxidized and native IgG, aggregates and monomeric species, as well as antibodies with mutations in the Fc and wild-type IgGs. Moreover, the chromatographic behavior of IgGs was shown to correlate with changes in the PK profile of FcRn transgenic mice.[63,64]

**Fig 9:**
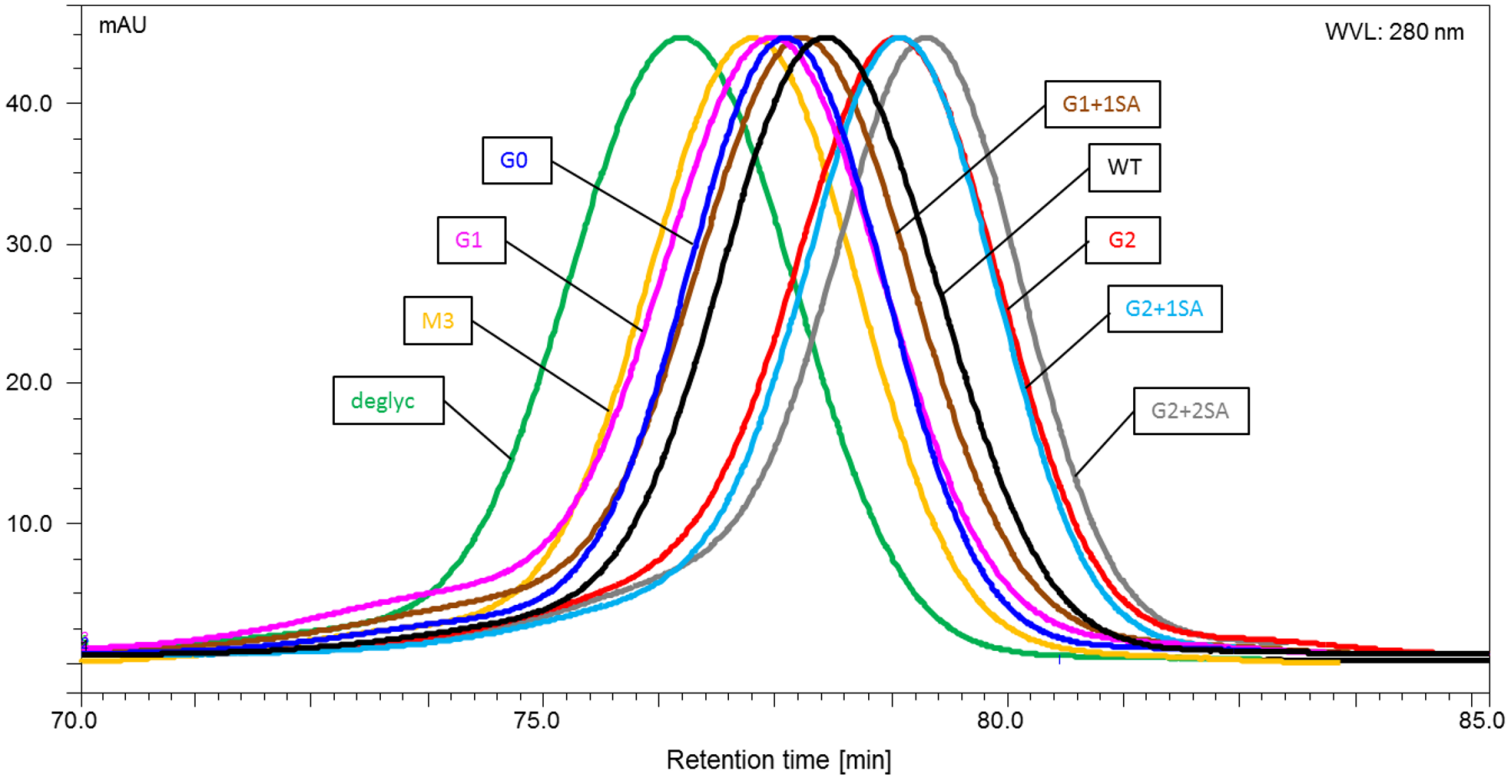
Retention profiles of IgG1 glycovariants obtained by analytical FcRn affinity chromatography.

Analyzed by FcRn chromatography, the deglycosylated IgG variant demonstrated decreased binding and eluted first, followed by M3, G1, G0, and G1+SA. The FcRn interaction was improved for the bi-galactosylated antibodies; moreover, the bi-sialylated variant demonstrated a slight shift to longer retention time, consistent with this variant having the strongest interaction (Fig 9).

Although the interaction of FcRn with antibodies has been reported to be independent of glycosylation,[65–68] SPR-analysis and FcRn affinity chromatography reported here, agree and show that the FcRn interaction can be influenced to some extent by the glycosylation pattern.

**Fig 10:**
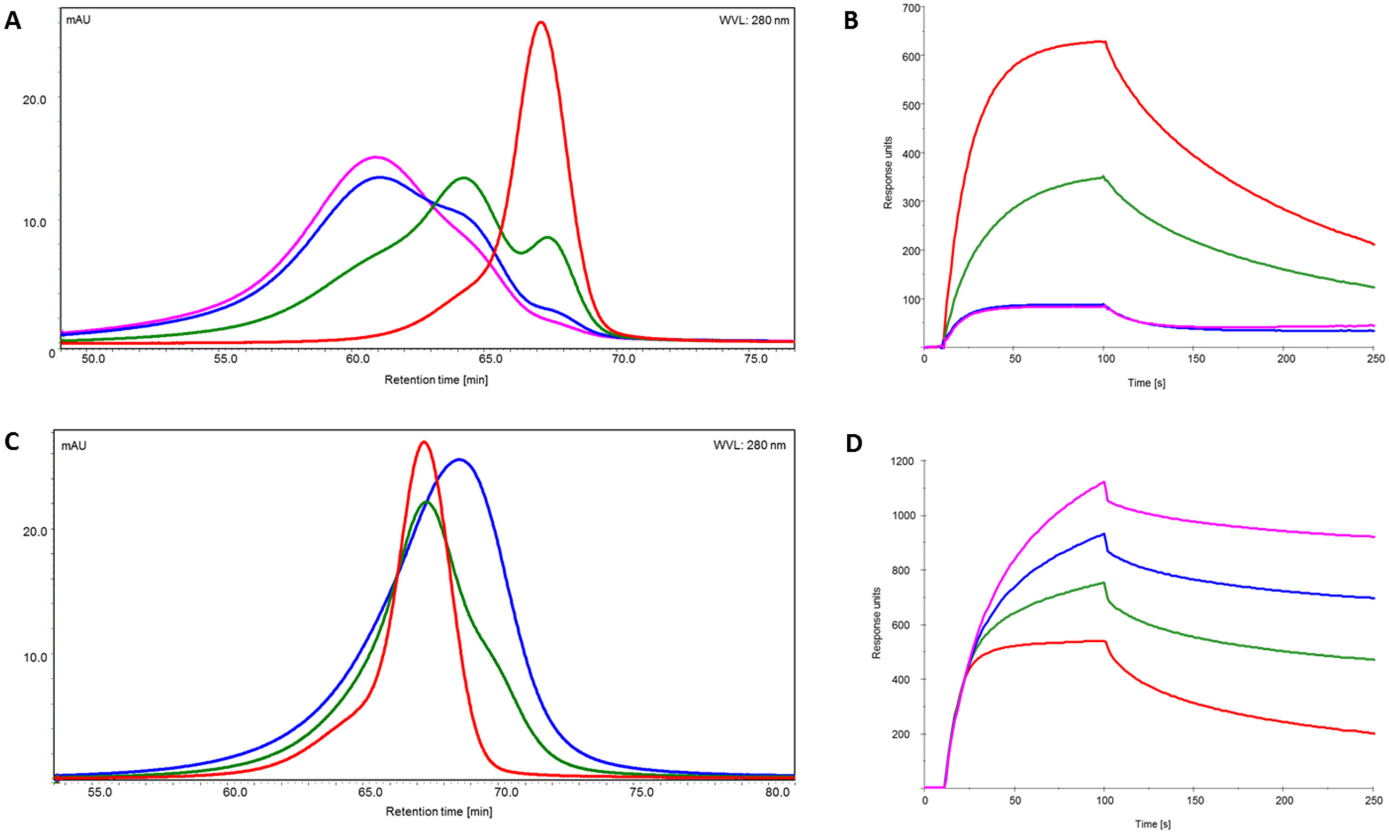
Interaction of target bound IgG with FcRn obtained by affinity chromatography and SPR. A. Retention profiles of IgG incubated in different ratios with its monomeric target. C. Retention profiles of IgG incubated in different ratios with F(ab’)_2_ <Fab>. B and D SPR analysis of interaction of IgG incubated with monomeric target and F(ab’)_2_ <Fab> respectively. Antibody: ligand ratio: 1:1 (green), 1:2 (blue), 1:4 (pink), IgG alone (red).

Analytical FcγRIIIa affinity chromatography and SPR revealed that the interaction of the antigen bound antibody with FcγRs, can be influenced by antigen properties, like its oligomeric state and its ability to induce formation of ICs. The FcRn affinity column was employed to examine interaction of target bound antibody and ICs with the receptor. IgG1 was again pre-incubated in different ratios with monomeric target and loaded onto the column. Analysis of the retention profiles showed three distinct antibody species: antigen-free, mono- and bivalently bound antibodies. Bivalently bound antibody had the weakest binding and eluted first, followed by the monovalently bound antibody fraction. Antigen free antibody demonstrated the strongest interaction with the immobilized FcRn and the longest retention time (Fig 10A). For the antibodies incubated with F(ab’)_2_<Fab> similar to the FcγRIIIa affinity column analysis, the interaction with FcRn was improved, and a slight shift to longer retention times could be observed (Fig 10C). In addition, the interaction of target-bound antibodies with FcRn was analyzed by SPR. Here, FcRn was immobilized on the chip surface, antibody incubated with monomeric target or F(ab’)_2_ <Fab> were subsequently injected as analytes. As with the FcγRIIIa, antibodies bound to monomeric target revealed decreased receptor interaction (Fig 10B), ICs created by the introduction of F(ab’)_2_ <Fab> demonstrated both an enhanced binding to the receptor and different association and dissociation behavior, compared to target free antibody (Fig 10D).

## Summary and Discussion

Interaction of IgG constant region with different elements of immune system is essential for antibody-mediated effector functions and its therapeutic efficacy. The IgG glycosylation pattern was demonstrated to have a crucial influence on the effector functions of therapeutic antibodies, and to contribute to structural and conformational stability of these proteins. [69–73] In this work, we systematically analyzed eight enzymatically engineered IgG1 glycovariants, highly enriched for the respective glycosylation pattern, with regard to effector functions and structural properties.

It has been reported for several antibodies that removal of the Fc-attached sugar moiety results in decreased stability of these mAbs. [74–79] In line with these reports, the deglycosylated antibody examined here demonstrated reduced thermal stability and a lower melting temperature than the remaining variants. M3 is another antibody glycovariant showing decreased thermal stability. The other variants examined demonstrated similar melting temperatures and no significant loss of monomer was observed for these thermally treated antibodies.

Binding to Fc gamma receptors is required for antibodies to initiate various immune responses, i.e. effector functions. Comparison of binding activities obtained with different experimental approaches demonstrated a recurring binding pattern. Consistent with previous reports, the deglycosylated antibody failed to bind all FcγRs except the high affinity FcγRI. The remaining glycovariants demonstrated gradually increasing binding affinities from the M3 to the G2-variant. M3, G0 and G1 interacted with the Fc receptors in an analogous manner and demonstrated decreased binding, compared to the WT antibody. Bi-galactosylated variants showed overall increased affinities, independent of experimental setup. This observation is in line with previous reports, showing a positive impact of terminal galactose residues on receptor interaction.[80–83] Moreover, it has been suggested that the fully galactosylated IgG-Fc adopts an "open" conformation, which may be optimal for the FcγR binding. [84] Impact of terminal sialic acid on the functionality of antibodies remains a controversial issue. Sialylated IgGs were reported to be involved in the anti-inflammatory pathway, and to have a decreased binding to FcγRs. For example, it was demonstrated that sialic acid bearing mouse IgG1 had a 10-fold decreased affinity towards activating receptors. [85] Sialylated antibodies are believed to be the pro-active species in IVIG (Intravenous Immune Globulin), which suppresses autoantibody-mediated inflammation by activating the inhibitory FcγRIIb and downstream negative signaling pathways. [86] However, this notion is still being debated. For instance, it has been demonstrated that the activating FcγRIIIa also plays an inhibitory role in mediating suppression of activating signaling and thus contributing to the anti-inflammatory properties of IVIG[87]. In addition anti-inflammatory activity of IgG1 was observed to be independent of sialylation and mediated by galactosylation, which induced cooperative signaling of the FcγRIIb with Dectin-1, resulting in an inhibitory signaling pathway that hindered pro-inflammatory effector functions. [88] Thus the role and impact of sialylation still need to be determined. Our analysis revealed no negative impact of sialic acid on interaction with the Fc–receptors; in contrast, these glycovariants demonstrated tendency to increased binding to both activating FcγRIIa and inhibitory FcγRIIb.

In addition to SPR analysis, FcγRIIIa affinity chromatography was employed to analyze the interaction of IgG with this receptor. As demonstrated previously, application of FcγRIIIa chromatography allows separation of the antibodies based on their affinity to the receptor (e.g. fucosylated and afucosylated IgGs [46], and provides an insight into homo-/heterogeneity of the analyzed sample. Analysis of glycovariants using an FcγRIIIa affinity column gave a binding pattern, similar to that obtained by SPR; i.e. increase in binding activity from M3 to G2 glycovariants. Remarkably, the binding pattern is consistent for fucosylated and afucosylated antibodies, suggesting that the remaining sugars contribute to Fc-mediated functions. A positive influence of terminal galactose residues could also be observed for the interaction of IgG1 glycovariants with FcγRIIIa expressing cells.

Altogether the results presented here demonstrate that composition of the Fc-attached sugar has a distinct impact on the antibody-receptor interaction. It should be pointed out that although our comparative analysis demonstrated enhanced binding affinities for bi-galactosylated and/or sialylated variants to the FcγRs, these results should be interpreted carefully. These positive effects are of a small magnitude, when compared to the impact of afucosylation on FcγRIIIa interaction (50 fold higher affinity) and ADCC.

In vitro analysis of the interaction of IgGs with Fc-receptors, which addresses the interaction of single antibodies with isolated Fc-binding domains of FcγRs, is unquestionably an important tool for an insight into the antibody—receptor interaction. However, the majority of FcγRs require immune complexes to fulfill their effector functions and initiate immune responses. [89–91] Here, we demonstrate that antibodies bound to their soluble monomeric targets have a dramatically impaired binding to FcγRIIIa. On the other hand, when we introduced F(ab)_2_<Fab> to mimic a bivalent target and to trigger IC formation, the interaction of these ICs with the receptor was improved. The decrease in affinities caused by target binding was compensated for by the avidity effect. These results suggest that the abundance and properties of the antigen is of great importance for immune responses. That is, a single antibody bound to its target might fail to trigger an effector function and thus formation of immune complexes is indispensable for Fc-mediated functionality.

FcRn is not only important for the transmission of neonatal immunity, but also determines the longevity of IgGs in serum and contributes to processing of immune complexes for subsequent antigen presentation. [12,65,92] Interaction of the FcRn with IgGs was reported to be independent of the antibody oligosaccharide pattern. [93] Comparison of binding activities of IgG1 glycovariants to FcRn obtained by SPR did not reveal any striking differences in binding activities, however bi-galactosylated antibodies showed slightly increased and deglycosylated IgG1 slightly decreased receptor interaction. Although the glycovariants analyzed here demonstrated differential FcRn interaction, it should be noted that these differences should be interpreted rather as the ability of the receptor to distinguish between different structural features, which do not necessarily reflect the pharmacokinetic properties of the antibody; e.g. it was demonstrated by Leabman et al. [94], that there was no difference in clearance between WT and deglycosylated IgG.

Analysis of the interaction, between target-bound antibodies and FcRn demonstrated a binding behavior similar to that obtained for the FcγRIIIa interaction. Soluble monomeric targets decreased binding affinities, whereas interaction of immune complexes with receptor was clearly improved. It is been recognized, that Fab–domains contribute to the antibody receptor interaction along with the Fc-region. Antibodies with identical Fcs but different Fab-domains were reported to differ in interaction with the FcRn, both in binding at acidic pH and dissociation at neutral pH, and as a result in pharmacokinetic properties. [95] Binding of monomeric target might hinder the formation of an optimal interaction interface, probably due to shielding of charges or some steric effects, which resulted in reduced receptor interaction. Formation of ICs leads to an avidity effect, which appears to overcome decreased affinity of target bound antibody. In contrast to the FcγRIIIa, where formation of immune complexes appears to be crucial for functionality, in the case of FcRn, increased avidity might lead to the opposite effect: the stronger interaction may obstruct the pH-dependent antibody binding and release, which may result in faster degradation of such ICs.

In summary, our multi angle functional analysis of eight enzymatically engineered glycovariants revealed a clear impact of Fc-glycosylation pattern on the interaction with FcγRs. In all assays we observed increasing binding activity from M3 to G2 glycovariants. This tendency also held true for the respective IC-functionality. Additionally, we observed that monomeric soluble target decreased IgG-receptor interaction, whereas every immune complex demonstrated an increased FcγR binding due to avidity effect, compared to any single IgG—Fc receptor interaction.

## Materials and Methods

### Production of IgG1 glycovariants

IgG1 glycovariants G0, G2, G2+1SA and G2+2SA were enzymatically engineered as described elsewhere. [46]

To obtain the G1+1SA variant, 100 mg of the G2+1SA IgG1 (c = 4.75 mg/ml) were treated with 2 ml of the β(1–4)-Galactosidase (Prozyme, GKX-5014) to cleave the galactose from the non sialylated branch of the glycan. The sample was incubated at 37°C for 48 h.

G1 glycovariant was produced by introduction of neuraminidase (Roche, 10269611001) to remove the remaining sialic acid from the G1+1SA glycan. 25 mg of the purified G1+1SA sample (c = 3.9 mg/ml) was desialylated. Therefore, 1.1 ml neuraminidase (11 units) in total were added to the sample in a stepwise manner over 14 h and incubated at 37°C.

To generate M3 glycovariant 5mg of G0 were treated with 1250 μl of β-N-acetylhexosaminidase (GKX-5003, Prozyme, 5U/100μl) at 37°C for 24 h.

Deglycosylated variant was produced from 15 mg of WT antibody by the addition of 120 μl N-Glycosidase F (Roche, 0.25 U/ml) and incubation for 24 h at 37°C.

The enzymatically generated glycovariants were purified by Protein A chromatography.

Molecule integrity was checked by LCMS peptide mapping and size exclusion chromatography. Glycan levels were determined by HPLC analysis after 2-AB labeling. Afucose levels (M5 not included) were determined by LCMS, as described in detail by Thomann et al.[46]. S1 Table.

### Isolation of Fc-fragments

To obtain Fc-fragments from full size IgGs, IgG glycovariants were digested with papain. For that 1/10 volume of 200 mM NaH_2_PO4, 10 mM EDTA, pH 6.3, 1/20 volume of 200 mM Cysteine and 15μg of papain per 1mg of antibody, were added to IgG solution. Mixture was incubated for 2h at 37°C. Fc-fragments were subsequently purified by protein A chromatography.

### Surface plasmon resonance (SPR) analysis

SPR interaction analysis was performed on a Biacore T200 system (GE Healthcare). For interaction analysis of captured FcγRs and IgG1 glycovariants, an anti-His capturing antibody (GE Healthcare) was injected to achieve a level of 12,000 resonance units (RU). Immobilization of the capturing antibody was performed on a CM5 chip using the standard amine coupling kit (GE Healthcare) at pH 4.5. 100 nM FcγRIa, 300 nM FcγRIIa_R131 and FcγRIIb, 200nM FcγRIIIa_V158 and 200nM FcRn were captured at respective concentrations with a pulse of 60 sec at a flow rate of 10 μl/min. Subsequently IgG1 glycovariants were applied at a concentration of 100–300 nM and a flow rate of 30 μl/min for 60 sec. The dissociation phase was monitored for 180 sec. The surface was regenerated by a 60 sec washing step with a 10 mM Glycine pH 1.5 at a flow rate of 30 μl/min.

For interaction analysis of the FcRs and target bound IgG1 glycovariants, IgG antigen was injected to achieve a level of 2,000 resonance units (RU). Immobilization of the antigen was performed on a CM5 chip using the standard amine coupling kit (GE Healthcare) at pH 4.5. IgG1 glycovariants were injected at a concentration of 50nM, with a pulse of 90 sec at a flow rate of 30 μl/min. Subsequently, 100 nM FcγRIa, 300 nM FcγRIIa, 300 nM FcγRIIb, 200 nM FcγRIIIa and 200 nM FcRn were applied at a flow rate of 30 μl/min for 90 sec. The dissociation phase was monitored for 180 sec. The surface was regenerated by a 60 sec washing step with a 10 mM Glycine pH 2 at a flow rate of 30 μl/min. For the interaction analysis with the F(ab’)_2_<Fab> or Protein L capturing, the latter were immobilized at pH 4.5, to achieve a level of 5000 RU, followed by the application of 50 nM IgG glycovariants. All experiments were carried out in HBS-N buffer (10 mM HEPES, pH 7.4, 150 mM NaCl). The Biacore T200 evaluation software was used for data evaluation.

### Analytical SEC / SEC MALLS

Analytical SEC was used to analyze aggregation propensity of the IgG1 glycovariants as well as to determine the size of antibody-antigen complexes. All experiments were carried out using a Dionex Summit system with UV detection at 280nm. Samples were analyzed with BioSuiteTM 250 column in 200mM K-Phosphate, 250 mM KCL, pH 7.0 at a flow rate of 0.5 ml/min at 20°C. SEC -MALLS experiments were performed on the Superose 6 column at a flow rate of 0.5 ml/min, under the same buffer conditions.

### Analytical FcγRIIIa affinity column

Biotinylated human FcγRIIIa_V158 was incubated with streptavidin sepharose for 2 hours upon mild shaking. The receptor derivatized sepharose was packed in a Tricorn 5/50 Column housing (inner diameter 5 mm x length 50 mm, GE Healthcare). Subsequently the affinity column was equilibrated with 20 mM Sodium Citrate, 150 mM NaCl pH 6.0 at flow rate of 0.5 ml/min using a Dionex Summit system. The antibody samples containing 30 to 50μg in equilibration buffer were loaded onto the column, washed with 5 column volumes of equilibration buffer and eluted with a linear pH gradient of 15 column volumes with 20 mM Citrate, 150 mM NaCl pH 3.0. The experiments were carried out at room temperature. The elution profile was obtained by continuous measurement of the absorbance at 280 nm.

### FcRn affinity chromatography

Antibody samples containing 50 to 150 μg of protein were adjusted to pH 5.5 and applied to the FcRn column using ÄKTA explorer 10 XT or Dionex Summit (Dionex). The column with 5 cm bed height was then washed with 5–10 column volumes of equilibration buffer 20 mM MES, 150 mM NaCl, pH 5.5. The affinity-bound Fc-containing proteins were eluted with a pH gradient to 20 mM Tris/HCl, 150 mM NaCl, pH 8.8, in 30 column volumes. Thereby, chromatography using FcRn columns mimicked physiological conditions with binding at acidic pH in the acidified endosome (pH 5.5–6.0) and release at pH 7.4 in the blood. For complete elution of modified antibodies, the pH is increased in the gradient up to pH 8.8. The experiments were performed at room temperature. The elution profile was obtained by continuous measurement of the absorbance at 280 nm. The time taken for an analyte peak, X, to reach the maximum detector signal after sample injection was called the retention time.

### Cell based FRET: binding to cells expressing huFcγRIIIa

Kit and reagents for the cell-based FRET assay were acquired from CisBio (www.cisbio.com). Briefly, 10,000 cells per well expressing huFcγRIIIa_V158-Tb were incubated with 50 nM IgG-d2 (provided in the kit) and decreasing concentrations of competing antibody (750–0.18 nM final concentration) at room temperature (RT). Emission intensity was measured at 620 nm and 665 nm at different time points with an Infinite M1000 PRO and the 665/620 ratio was calculated after background subtraction (cells only).

## Supporting Information

S1 Fig SEC retention profiles of IgG glycovariants. IgG glycovariants were incubated 10 days at 37°C and pH 6.0 (A) or pH 7.4 (B). The antibody main peak has a retention time of 16 to 17 min. Formation of oligomeric species (retention time 13.5–15min) could be observed for all glycovariants in a range of 2–3% at both pH conditions.
(TIF)

S2 Fig SPR analysis of interaction of IgG glycovariants with FcγRs. A. SPR analysis of interaction of Fc fragments of IgG glycovariants with FcγRs. Anti His-antibody immobilized on the chip, glycovariants were injected as analytes, after capturing of the respective receptors: FcγRI (black), FcγRIIa (grey), FcγRIIb (dark grey) and FcγRIIIa (light grey). Binding of WT Fc-fragment was set as 100%. Each graph represents results from at least three independent experiments; data are given as means ± SD. B. IgG1 WT binding to immobilized FcyRIIa. Here IgG1WT have been titrated in a concentration series of 8000nM in 1:1 dilutions down to 32nM, allowing a global Rmax calculation. The applied concentration should allow a saturation of the FcyRIIa. The black fitting curve only describes a concentration dependent bulk effect without a visible saturation. Therefor this evaluation has been regarded as not suitable for the evaluation of different glycosylation profiles of the tested antibody. C. IgG1 WT binding to immobilized FcyRIIIa. Here IgG1WT have been titrated in a concentration series of 8000nM in 1:1 dilutions down to 32nM, allowing a global Rmax calculation. The applied concentration should allow a saturation of the FcyRIIIaV158 receptor. The black fitting curve does not describe the measured curves for the three highest concentrations at all. Similar problems occur if possible bulk contributions were not allowed. Thereby a 1:1 kinetic evaluation is not applicable in this case.
(TIF)

S3 Fig Binding of glycovariants to FcγRIIIa, expressed on living cells. Unlabeled glycovariants compete for binding to receptor with acceptor-labeled antibody, resulting in decrease of FRET signal. Initial signal was normalized to 1.
(TIF)

S4 Fig SEC retention profiles of IgG incubated with monomeric target and F(ab’)_2_<Fab>. IgG1 was incubated with monomeric target in different ratios; IgG:target: 0:1 (black) 1:0 (red), 1:1 (green), 1:2 (blue) and 1:4 (pink) (A) and with F(ab’)_2_ <Fab>; IgG: F(ab’)_2_<Fab> 1:0 (black), 1:1 (blue) (B).
(TIF)

S5 Fig Comparative SPR analysis of interaction of single and F(ab’)_2_ <Fab> linked IgG glycovariants with FcγRIIIa. FcγRIIIa was captured by anti His-antibody immobilized on the chip, followed by application of single IgGs (light grey) and F(ab’)_2_ <Fab> linked IgG glycovariants (dark grey). IgG was incubated with F(ab’)_2_ <Fab> in 1:1 ratio.
(TIF)

S6 Fig SPR analysis of interaction of F(ab’)_2_ <Fab> linked IgG1 with FcγRIIa and FcγRIIb. IgG1 was incubated in different ratios with F(ab’)_2_ <Fab> and loaded onto the captured FcγRIIa (A,B) and FcγRIIb (C,D). IgG:ligand ratio: 1:1 (green), 1:2 (blue), 1:4 (pink), IgG alone (red).
(TIF)

S1 Table Results of in-vitro glycoengineering. List of the relative occurrence of Glycan Species [%].
(TIF)

## Abbreviations

ADCC: antibody-dependent cell-mediated cytotoxicity
CDR: complement determining region
CHO: chinese hamster ovary
Fab: fragment antigen binding
Fc: fragment crystallizable
FcR: Fc receptor
FcγRs: Fc gamma receptors
FcRn: neonatal Fc receptor
HPLC: high performance liquid chromatography
IC: immune complex
mAb: monoclonal antibody
SA: sialic acid
SEC: size exclusion chromatography
SPR: surface plasmon resonance
TSA: thermofluor stability assay
