# Supplementary figures and images for: Multi-Angle Effector Function Analysis of Human Monoclonal IgG Glycovariants
Source: PLoS One. 2015 Dec 11;10(12):e0143520. doi: 10.1371/journal.pone.0143520 (PMC4676693; doi:10.1371/journal.pone.0143520)

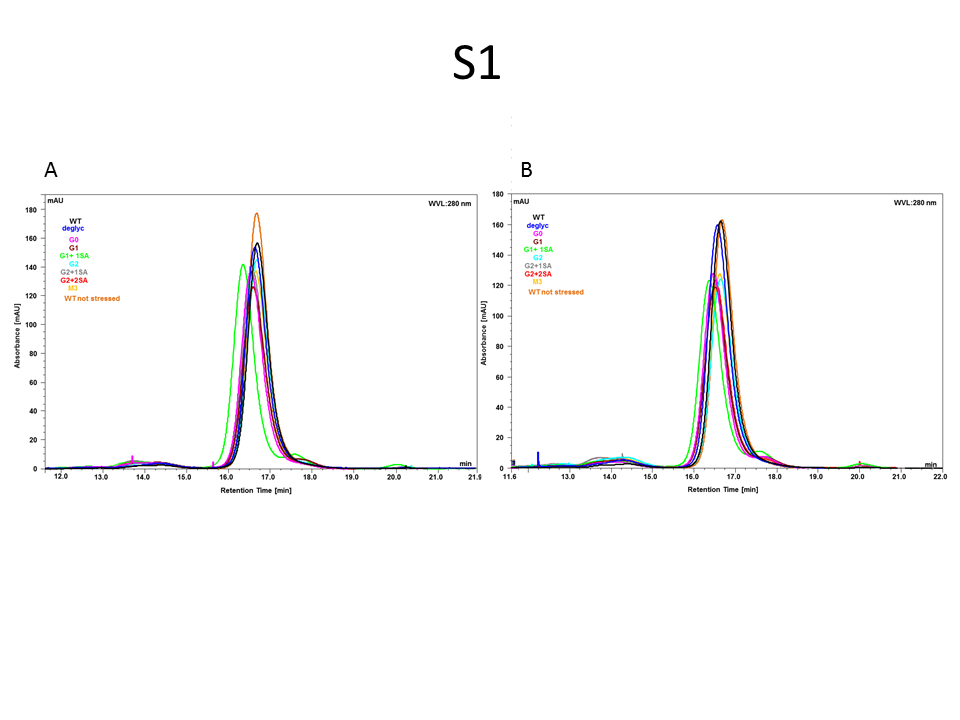

Supplement: S1 Fig — IgG glycovariants were incubated 10 days at 37°C and pH 6.0 (A) or pH 7.4 (B). The antibody main peak has a retention time of 16 to 17 min. Formation of oligomeric species (retention time 13.5–15min) could be observed for all glycovariants in a range of 2–3% at both pH conditions. (TIF) [file pone.0143520.s001.tif]

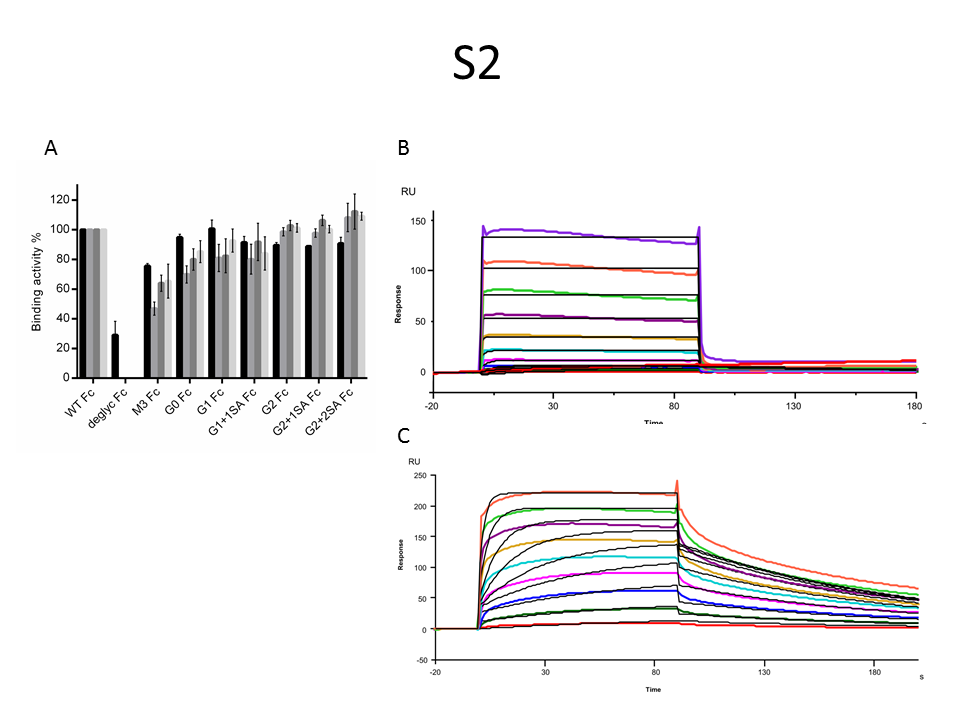

Supplement: S2 Fig — A. SPR analysis of interaction of Fc fragments of IgG glycovariants with FcγRs. Anti His-antibody immobilized on the chip, glycovariants were injected as analytes, after capturing of the respective receptors: FcγRI (black), FcγRIIa (grey), FcγRIIb (dark grey) and FcγRIIIa (light grey). Binding of WT Fc-fragment was set as 100%. Each graph represents results from at least three independent experiments; data are given as means ± SD. B. IgG1 WT binding to immobilized FcyRIIa. Here IgG1WT have been titrated in a concentration series of 8000nM in 1:1 dilutions down to 32nM, allowing a global Rmax calculation. The applied concentration should allow a saturation of the FcyRIIa. The black fitting curve only describes a concentration dependent bulk effect without a visible saturation. Therefor this evaluation has been regarded as not suitable for the evaluation of different glycosylation profiles of the tested antibody. C. IgG1 WT binding to immobilized FcyRIIIa. Here IgG1WT have been titrated in a concentration series of 8000nM in 1:1 dilutions down to 32nM, allowing a global Rmax calculation. The applied concentration should allow a saturation of the FcyRIIIaV158 receptor. The black fitting curve does not describe the measured curves for the three highest concentrations at all. Similar problems occur if possible bulk contributions were not allowed. Thereby a 1:1 kinetic evaluation is not applicable in this case. (TIF) [file pone.0143520.s002.tif]

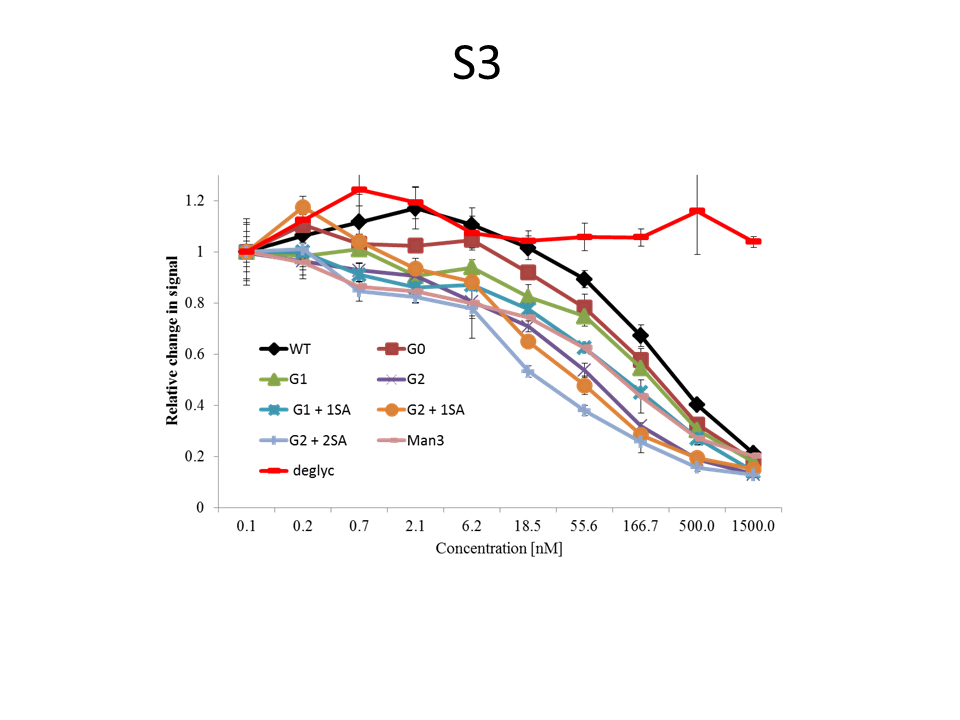

Supplement: S3 Fig — Unlabeled glycovariants compete for binding to receptor with acceptor-labeled antibody, resulting in decrease of FRET signal. Initial signal was normalized to 1. (TIF) [file pone.0143520.s003.tif]

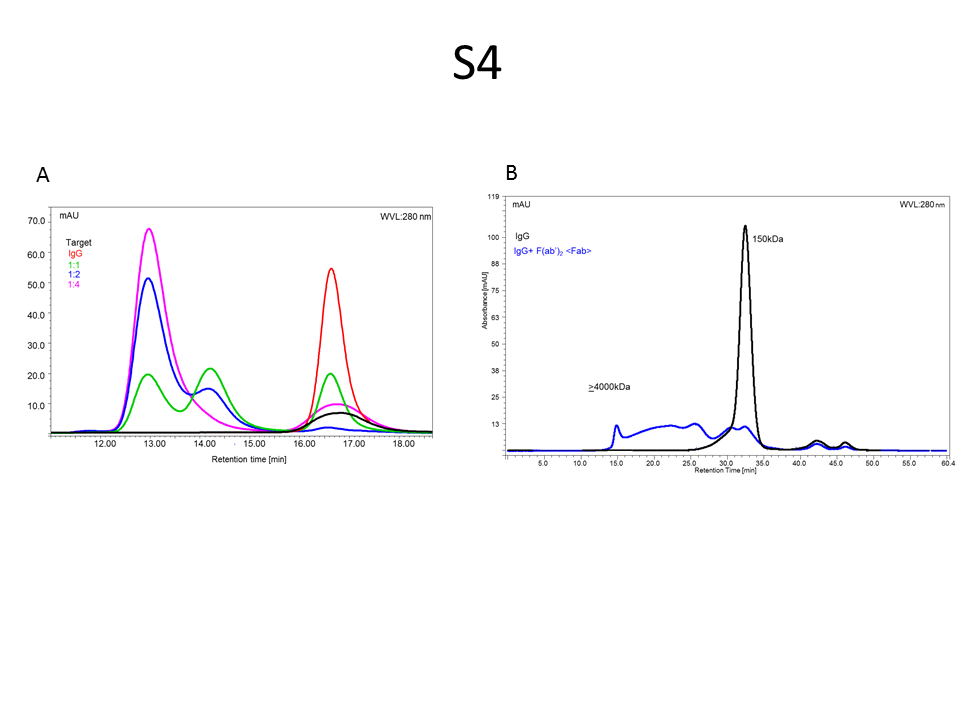

Supplement: S4 Fig — IgG1 was incubated with monomeric target in different ratios; IgG:target: 0:1 (black) 1:0 (red), 1:1 (green), 1:2 (blue) and 1:4 (pink) (A) and with F(ab’)2 ; IgG: F(ab’)2 1:0 (black), 1:1 (blue) (B). (TIF) [file pone.0143520.s004.tif]

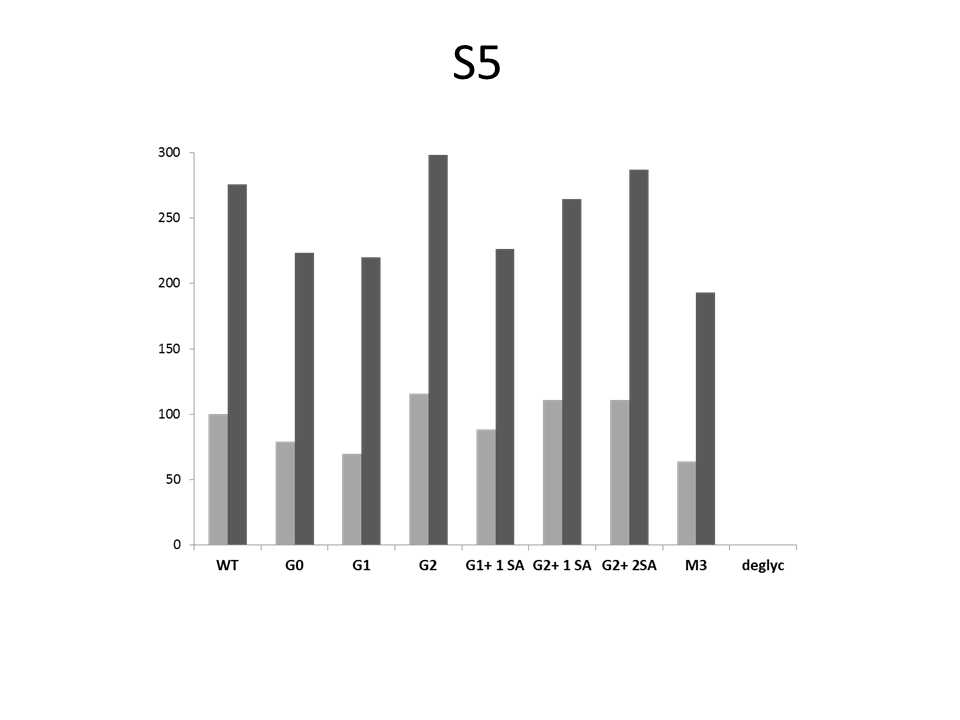

Supplement: S5 Fig — IgG was incubated with F(ab’)2 in 1:1 ratio. (TIF) [file pone.0143520.s005.tif]

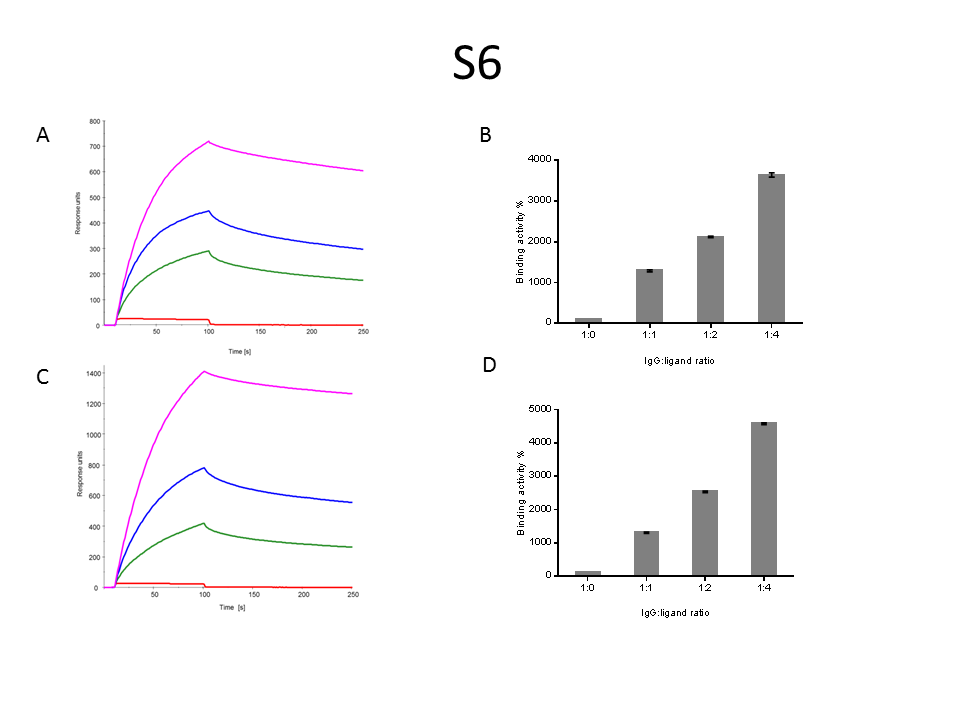

Supplement: S6 Fig — IgG1 was incubated in different ratios with F(ab’)2 and loaded onto the captured FcγRIIa (A,B) and FcγRIIb (C,D). IgG:ligand ratio: 1:1 (green), 1:2 (blue), 1:4 (pink), IgG alone (red). (TIF) [file pone.0143520.s006.tif]

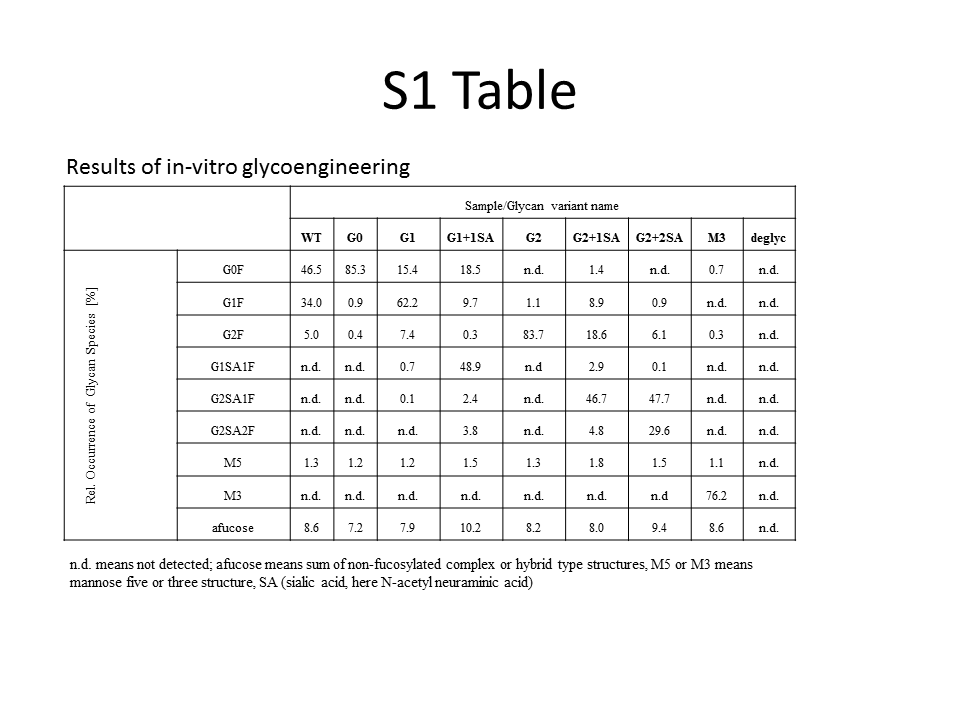

Supplement: S1 Table — List of the relative occurrence of Glycan Species [%]. (TIF) [file pone.0143520.s007.tif]
